# Molecular drivers and cells of origin in pancreatic ductal adenocarcinoma and pancreatic neuroendocrine carcinoma

**DOI:** 10.1186/s40164-020-00184-0

**Published:** 2020-10-22

**Authors:** He-Li Gao, Wen-Quan Wang, Xian-Jun Yu, Liang Liu

**Affiliations:** 1 Department of Pancreatic Surgery, Fudan University Shanghai Cancer Center, 270 Dong An Road, Shanghai, 20032 People’s Republic of China; 2 Department of Oncology, Shanghai Medical College, Fudan University, 270 Dong An Road, Shanghai, 200032 People’s Republic of China; 3 Shanghai Pancreatic Cancer Institute, 270 Dong An Road, Shanghai, 200032 People’s Republic of China; 4 Pancreatic Cancer Institute, Fudan University, 270 Dong An Road, Shanghai, 200032 People’s Republic of China

**Keywords:** Pancreatic adenocarcinoma, Pancreatic neuroendocrine carcinoma, Neuroendocrine tumor, Carcinogenesis, Genomic patterns

## Abstract

Pancreatic cancer is one of the most common causes of cancer-related deaths worldwide. The two major histological subtypes of pancreatic cancer are pancreatic ductal adenocarcinoma (PDAC), accounting for 90% of all cases, and pancreatic neuroendocrine neoplasm (PanNEN), which makes up 3–5% of all cases. PanNEN is classified into well-differentiated pancreatic neuroendocrine tumor and poorly-differentiated pancreatic neuroendocrine carcinoma (PanNEC). Although PDAC and PanNEN are commonly thought to be different diseases with distinct biology, cell of origin, and genomic abnormalities, the idea that PDAC and PanNEC share common cells of origin has been gaining support. This is substantiated by evidence that the molecular profiling of PanNEC is genetically and phenotypically related to PDAC. In the current review, we summarize published studies pointing to common potential cells of origin and speculate about how the distinct paths of differentiation are determined by the genomic patterns of each disease. We also discuss the overlap between PDAC and PanNEC, which has been noted in clinical observations.

## Introduction

Pancreatic ductal adenocarcinoma (PDAC) is the most common type of malignancy found in the pancreas, with over 90% of pancreatic neoplasms diagnosed as PDAC. Epidemiologic studies of all malignant types have shown that the 5-year relative survival rate is the lowest in PDAC and that death rates from this type of cancer have been rising over the past decade [1]. Less than 5% of patients with PDAC are alive after 5 years, and in patients discovered in the early stages, the 5-year survival rate is only approximately 20% [2]. The most common driver gene mutations of PDAC include KRAS, CDKN2A, TP53, and SMAD4, which all together account for 90% of cases [3].

The second most common type of pancreatic neoplasm is pancreatic neuroendocrine neoplasm (PanNEN), which accounts for 3–5% of all cases [4]. It is important to note that poor differentiation of pancreatic neuroendocrine carcinoma (PanNEC) accounts for 10–20% of PanNEN cases. PanNEN has a prognosis similar to PDAC, with a median overall survival (OS) of only 7.5 months [5]. In contrast, well-differentiated pancreatic neuroendocrine tumors (PanNETs) are slow-growing with 5- and 15-year OS rates of 85.4% and 55%, respectively. In addition to distinguished clinicopathological characteristics and prognosis, PanNEC tumors are known to be histologically and genetically different from PanNET. For example, common somatic mutations in PanNET include MEN1 (multiple endocrine neoplasia type 1), DAXX (death-domain-associated protein)/ATRX (alpha alassemia/mental retardation syndrome X-linked), and mTOR (mammalian target of rapamycin) pathway genes. However, PanNEC tumors carry the mutations of tumor protein 53 (TP53), retinoblastoma 1 (RB1), and KRAS [6], which is similar to what is seen in PDAC, while differing completely from what occurs in PanNET [7].

The close association between adenocarcinoma and neuroendocrine carcinoma has been shown in other organs, such as the colon and rectum [8]. Transformation from non-small cell lung cancer to small cell lung cancer is commonly encountered in clinical practice, and supports the idea that these malignancies might share common cells of origin [9]. The current 2019 World Health Organization (WHO) TNM classification for NEN indicates that high-grade PanNEC should be classified according to the criteria used for classifying the carcinomas of the pancreas [10]. In addition, in clinical practice the standard chemotherapy for PDAC is also effective in treatment of PanNEC [11]. This raises the question of whether PDAC and PanNEC may have deeper connections. In this review, we summarize connections between PDAC and PanNEC including their genomic features, cells of origin, and clinical practice areas.

## Embryo development and molecular regulation of pancreatic exocrine and endocrine secretions

The pancreas is an organ with dual endocrine (hormone-producing) and exocrine (enzyme-producing) functions. Exocrine secretions are formed with acini and ducts and secrete digestive proenzymes into the duodenum where they aid the digestion process. Exocrine secretion, which is represented by the islet of Langerhans, contains five distinct cell types (α-cells, β-cells, δ-cells, γ-cells, and ε-cells), and hormones, such as glucagon, insulin, and somatostatin, are secreted directly into blood circulation [12].

**Fig. 1:**
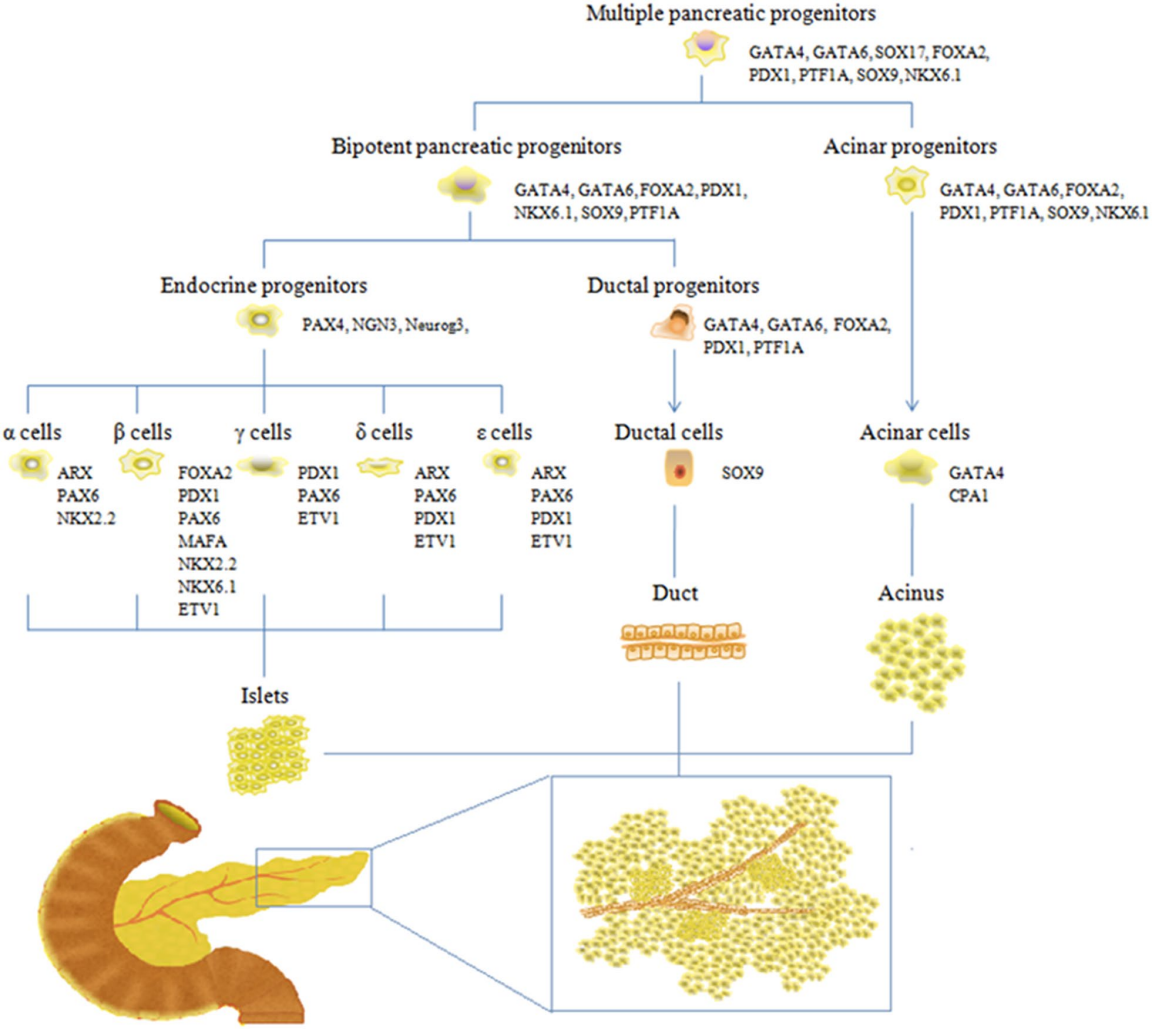
Endocrine and exocrine cell differentiation during pancreas development

During the process of embryo development, however, both endocrine and exocrine cells originate from a common pool of multipotent pancreatic progenitors, which subsequently differentiate into the acing progenitors and bipotent pancreatic progenitors (bi-PPs) (Fig. 1). The former differentiate into acinar cells, while the latter give rise to ductal progenitors and endocrine progenitors which further separate into ductal cells and endocrine cells, respectively [13]. Three major epithelial cell types (islet, acinar and duct) found in the pancreas develop from the same undifferentiated pancreatic progenitors, yet have radically different functions, which indicates an interesting mechanism. The mechanism seen in the differentiation process is not synchronous, as several molecules and pathways participate in this process (Fig. 1). GATA4, GATA6, FOXA2, and PTF1A are expressed in the foregut endoderm and play important roles in the differentiation of pancreatic progenitors [14, 15]. SOX9 persists in pancreatic duct cells, but it is absent in endocrine cells and acinar cells [16]. The mechanisms that initiate the formation of specific endocrine subtypes are more sophisticated. The most effective differentiation in α-lineage is observed in Aristaless-related homeobox (ARX)expressing cells, which effectively secrete glucagon [17]. Genes for β-cell differentiation and maintenance of function are multiple. Typical genes for functional β-cell differentiation are NEUROD1, INS1, MAFA, ISL1, PDX1, and ACVR1C [18]. Genetic markers that maintain mature β-cell function and identity include MAFA, PDX1, NKX2.2 and NKX6.1 [12, 16, 19]. Ductal cell lineages and endocrine cell lineages interact and exhibit common genetic differentiation during development of the pancreas.

### Genomic features shared among PDAC, PanNET, and PanNEC

**Fig. 2:**
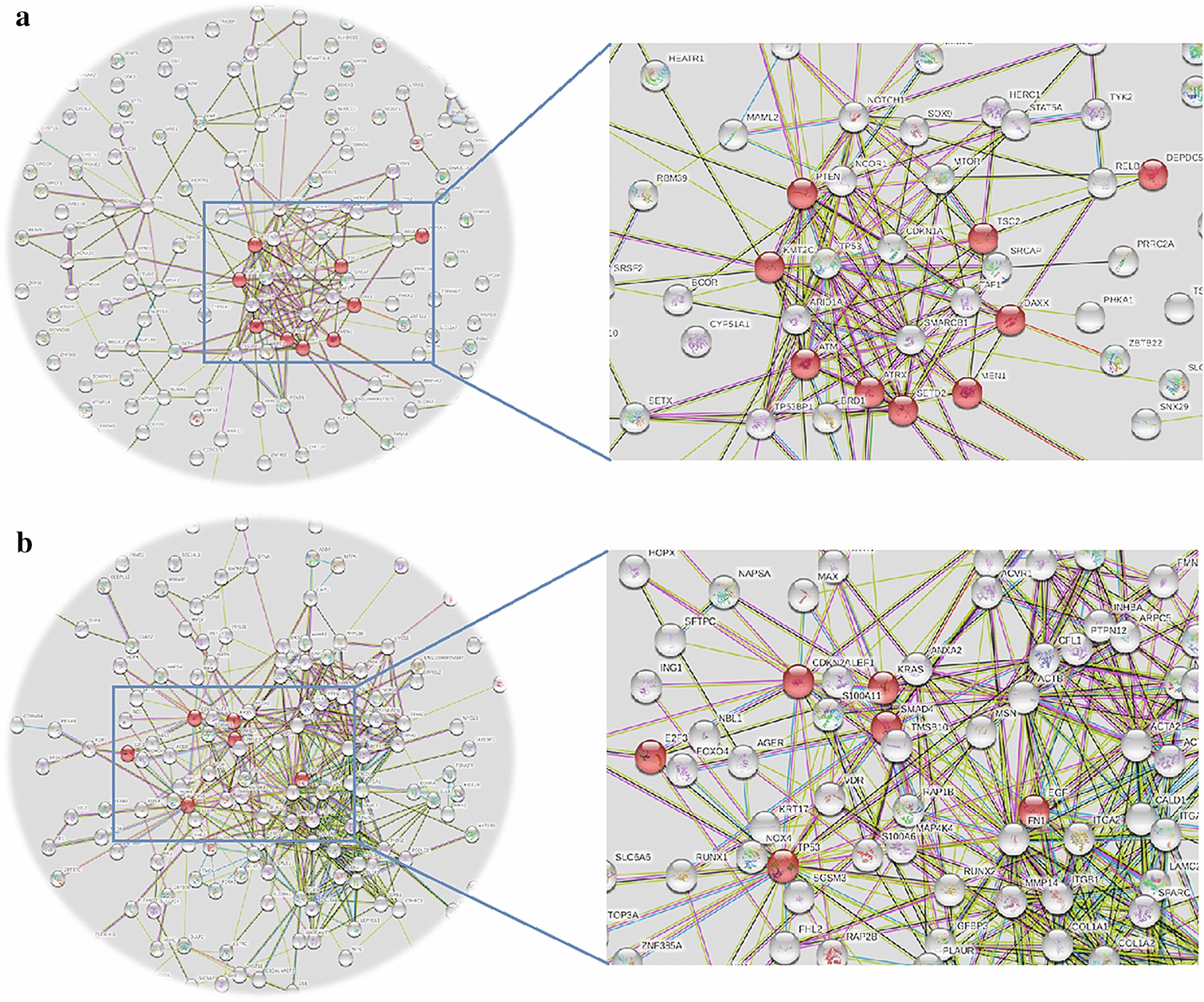
Driver gene association network between PanNET (**a**) and PDAC (**b**)

There are major differences in driver gene landscapes and related pathways among PDAC, PanNET and PanNEC (Fig. 2, Table 1). The most common driver genes found in PDAC are KRAS (88–100% of cases), TP53 (85% of cases), CDKN2A (90% of cases) and SMAD4 (55% of cases) [20, 21]. These four driver genes fulfill key roles in pancreatic tumorigenesis including the transition of pancreatic precursors such as PanIN and IPMN to PDAC [22]. In addition, about 10% of PDAC manifestations are inherited, and the best-studied germline mutations linked to familial pancreatic cancer risk are the components of DNA double-strand break repair machinery, including BRCA1, BRCA2, PALB2 (partner and localizer of BRCA2), the Fanconi anemia genes FANCC and FANCG, and ATM (ataxia telangiectasia mutated) [23].

**Table 1:** Summary of the core pathways and genes in PDAC, PanNET and PanNEC

Pathways	PDAC	PanNET	PanNEC
RAS/MAPK signaling	*KRAS, MAP2K4*		*KRAS, BRAF*
NF-kB signaling	*NF*-*κB, IKK*		
JAK/STAT signaling	*MAP4K3, TNF, BCL2*		*BCL2*
TGFb/SMAD signaling	*TGF*-*bR2, SMAD4*		
Wnt/b-catenin signaling	*DKK1, HMGA2*		*WNT*
Hedgehog signaling	*SOX3,GLI1,GLI3, TBX5, LRP2*		*LRP2*
Hippo signaling	*YAP, TAZ*		
SWI/SNF	*ARID1A*		
Small GTPase-dependent signaling	*DEPDC2*		
Notch signaling	*MYC, GATA6*	*NOTCH1, HES1*	
DNA damage repair	*TP53, BRCA1, BRCA2*	*TP53, MDM2, MDM4*	*TP53*
Cell cycle	*CDKN2A, APC2, FBXW7, CHD1*	*CDK4, p16,p27*	*RB1, APC, CDKN2A, FBXW7*
c-MET		*MET*	
PI3K/AKt signaling		*PTEN, PI3KCA, AKT, mTOR, TSC1/2*	*PTEN, PI3KCA, mTOR*
Chromatin remodeling		*DAXX,ATRX, MDM2*	
Angiogenesis pathway	*HIF*-*1, BNIP3*	*HIF*-*1a, VEGF, PDGF, PHDs,Kit, Tie*-*2*	
Menin pathway		*MEN1, MLL*	

Comparisons between genetic landscapes of PanNEC and PDAC indicate that almost half of PanNEC instances are genetically and phenotypically related to PDAC [24]. Whole exon analysis shows that TP53 and RB are the most common altered genes in NEC [25]. TP53 mutations are present in approximately 70–95% of PanNEC cases [26], and Rb markers have been identified in 74% of the instances of PanNEC [27]. Mutations in TP53 and RB1 genes are the pivotal drivers, and related pathways are central features of PanNEC development and are associated with poor survival [28]. Other predominantly mutated genes involve APC, CDKN2A, BRAF, KRAS, PTEN and PIK3CA [28, 29]. FBXW7, WNT, BCL2, and CTNNB1, which are mutated in partial PDAC, are also related to PanNEC oncogenesis but at a lower rate of occurrence [30]. Most KRAS-positive PanNEC also express MUC1 and carcinoembryonic antigen (CEA) as the markers of ductal differentiation. Exocrine lineage markers may help to reveal the potential relationship of some PanNEC occurrences with conventional PDAC. Activating KRAS mutations in PanNEC expression show the same hot spot areas in PDAC, such as exon 2 and exon 3 ++− [27]. The most common gene mutations in mixed neuroendocrine-nonneuroendocrine neoplasms (MiNENs) are TP53, KRAS, and BRAF [31], which have been found to overlap between PanNEC and PDAC. One study has shown that in MiNEN, the mutations of CDKN2A, GNAS, ERBB2, and BRAF found in the PanNEC section were located next to the PDAC section [32].

The driver genes in PanNETs are totally different from those found in PanNEC [33], PanNEC has a much higher mutation burden when compared with PanNETs. Next generation sequencing analysis has shown that the most common gene mutations in PanNETs include MEN1 (37%), DAXX/ATRX (22% and 10%), and mTOR pathway genes, particularly PTEN(7%), tuberous sclerosis complex 2 (TSC2, [4%]) and PIK3CA [7]. MEN1 acts as a hub gene and interacts with all core pathways, and DAXX/ATRX also cooperates during tumorigenesis [34, 35]. The NET G3 subgroup, defined in a recent WHO classification, has the similar genetic mutations as PanNET G1/2, namely ATRX(19%), SF3B1(19%), and MEN1(12%) [29]. However, MEN1 and DAXX/ATRX genes mutations seem to occur very rarely in PanNEC [36]. Whereas mutation rates in RB and TP53 appear to be very low in PanNETs. In general, 80% of PanNETs do not have mutations in the Rb protein [37]. Even in PanNET G3, RB abnormalities were lower than those in PanNEC G3 (42% to 71.4%) [26, 33]. Overexpression of the Bcl-2 protein was observed in 50–100% of PanNECs, higher than that found in PanNETs (18%). Furthermore, Bcl-2 overexpression was significantly correlated with higher Ki67 in PanNEC [38]. In addition, the PanNEC expression levels of mTOR were higher than those found in PanNETs [39]. Some genes that are often seen to mutate in PDAC can be found to be abnormal in PanNETs, but this always is a more aggressive characteristic, leading to poor prognosis. For example, high expression of Cdk4 has been shown to lead to inactivation in PanNETs and is associated with a higher grade [40].

Twelve classical signaling pathways accompany PDAC tumorigenesis. Among them, the Hedgehog pathway, Notch pathway, Wnt pathway, RAS/MAPK/PI3K pathway, and JAK-STAT pathway are recognized as main contributors to PDAC progression [41, 42]. Unlike PDAC, DNA damage repair, chromatin remodeling, telomere alteration, and the PI3K/mTOR pathway are main pathways in the PanNET process.

## Cells of origin that are shared among PDAC, PanNET, and PanNEC

The origins of PDAC and PanNEN are complex. PDAC can arise either from a precursor cell of intralobular ducts or acinar cells of exocrine secretion. The process of PDAC tumorigenesis is initiated by oncogenic KRAS and requires the repression of the epithelial differentiation program and activation of factors that are normally expressed during the embryonic development of the pancreas. Precursors of pancreatic cancer, namely pancreatic intraepithelial neoplasia (PanIN), intraductal papillary mucinous neoplasm (IPMN), and mucinous cystic neoplasm (MCN), participate in this process. Indeed, up to 33% of pancreatic tissue from autopsy series contains PanIN [43]. Duct-like cell lineages with a mutation in the driver gene KRAS, can develop into PanIN and progress to PDAC after additional multi-genetic (CDKN2A, SMAD4, and TP53) processes. The genetic features associated with IPMN and MCN are less well characterized [44]. Acinar cells transform to a duct-like phenotype during an acinar-to-ductal metaplasia (ADM) process. With a mutation in the driver gene KRAS, ADM cells stay locked in a ductal stage, and progress to PanIN and PDAC during additional multi oncogenic signaling [45, 46].

PanNETs are heterogeneous, and their differences can be explained partly by the PanNET cells of origin. Specifically, at least two different progenitor cells can give rise to non-functional PanNETs, the α-cells lineage (ARX +) or β-cells lineage (PDX +) [19]. Global DNAme profiles of the early stage of PanNET show strong similarities with both α- and β-cell DNAme profiles, while late stage tumors show a lower degree of similarities with normal cell types [47]. An additional study confirmed that different molecular subtypes of PanNET arise from different cellular origins, or from mature β-cell lineage, or islet cell precursors [48]. Loss of MEN1 is an early event in PanNET tumorigenesis, while ATRX/DAXX loss and ALT are relatively late events [49]. ACVR1C plays a key role in islet cell differentiation and also works as a suppresser during PanNET development. ACVRIC also contributes to a higher Ki67 index [50].

**Fig. 3:**
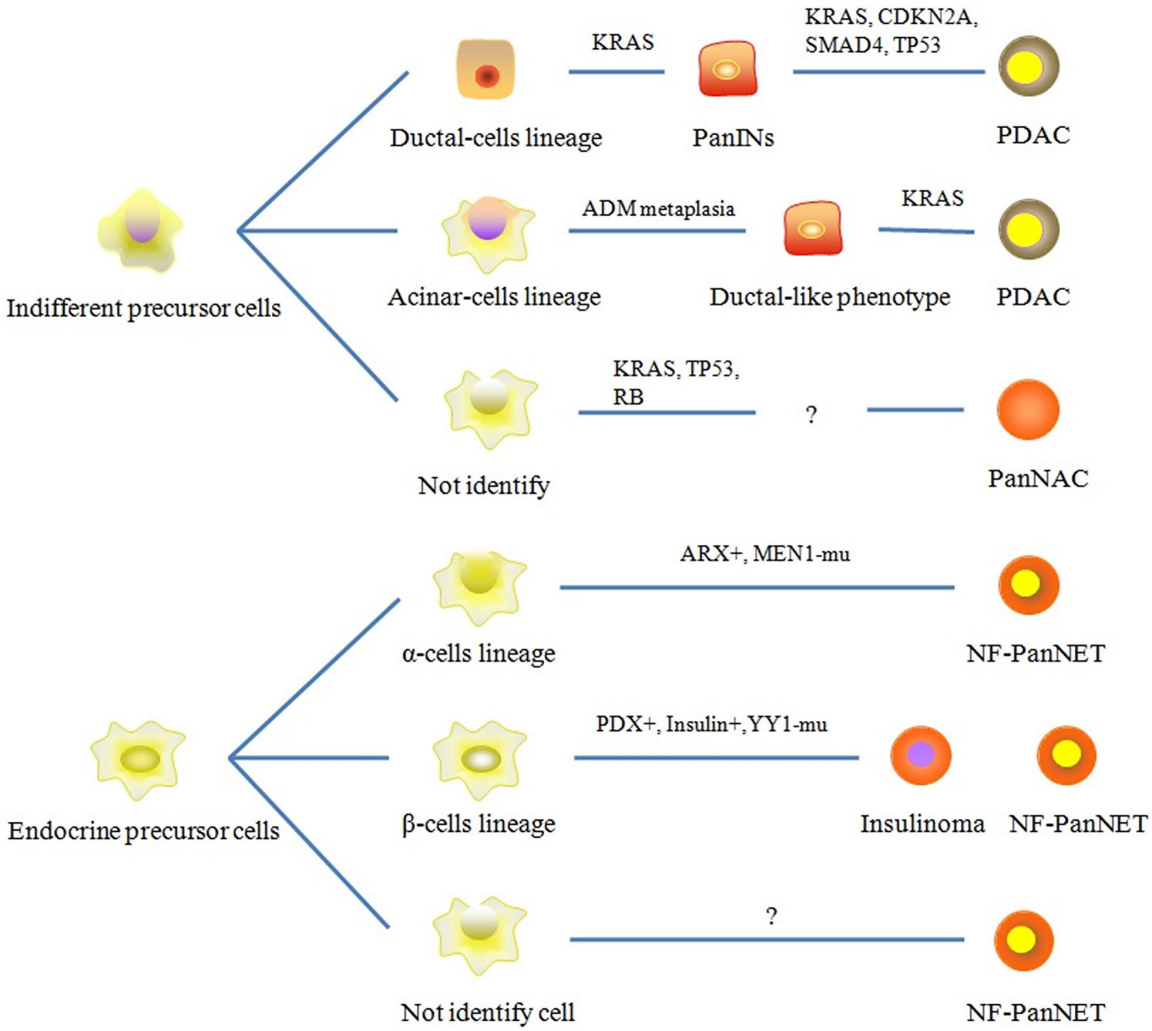
Hypothesis of the evolution of PanNET, PDAC, and PanNET cells of origin

PanNEC cell of origin is more sophisticated. PanNEC may in fact originate from a separate, potentially non-neuroendocrine lineage, as seen in the growing body of evidence showing that the genomic origination of PanNEC is different from that of PanNETs. PanNEC may originate from undifferentiated progenitor cells [51], which is the same in the occurrence of PDAC (Fig. 3). A mouse model of PanNEC has shown a loss of many markers during beta-cell, alpha-cell, and delta-cell differentiation, including markers of endocrine progenitors, such as MAFA, PDX1, and NKX6.1. RB and/or the p53 signaling network are associated with this process. Furthermore, Id1, a marker of neural stem cells, was identified as specifically expressed in PanNEC [52]. This raises the possibility that PanNEC may have stem cell-like properties or arise from pancreatic progenitor cells.

## Tumor microenvironment among PDAC, PanNET, and PanNEC

In addition to genetic landscape, the tumor microenvironments (TME) of these neoplasms also have distinct characteristics. PDAC has a unique desmoplastic TME, which generates a large stromal component and accounts for 15–90% of the tumor [53]. This desmoplastic stroma compromises immune cells, tumor blood perfusion and oxygen delivery, in turn generating an obstacle for angiogenesis. This identifies another feature of PDAC, impaired tumor vasculature and hypoxia [54]. Desmoplastic stroma causes obstacles in chemotherapy and anti-angiogenesis target therapy in PDAC. Most PanNEC cases exhibit hypo vascular patterns similar to PDAC. In contrast, PanNETs have extraordinary tumor vascularization with high expression of several pro-angiogenic molecules, including platelet-derived growth factor receptors and, vascular endothelial growth factor and its receptors [55]. The hyper vascular characteristics of PanNET provide a way to **t**arget angiogenesis therapy [56].

It has been observed that profiles of tumor-infiltrating lymphocytes (TILs) in PanNEN and PDAC samples form three distinct clusters and are concordant with histological types PanNEC, PDAC, and PanNETs [57]. This indicates a correlation between immune profile and tumor histology. PanNEC and in some instances, PDAC have revealed a hot immune TME when compared to PanNETs. TIL and its PD-1/PD-L1 + subsets have been found less abundant in PanNETs compared to those found in PanNEC and PDAC [57]. The stroma of PDAC is rich in CD3 + CD4 + helper T (Th) cells, CD8 + T cells, and CD4 + CD25 + fork-head box P3 regulatory T cells. PD-1 and PD-L1 are associated with poor prognosis in patients with PDAC [58]. PanNEC shows the intensive accumulation of CD3 +/CD4 T cells, which is comparable with that found in PDAC [57]. There is a clear shift at the NET–NEC transition in immune-related profiles of GEP-NEN. Reduced CD8 + infiltration and enhanced PD-L1 expression in tumor cells are associated with higher tumor grading [59]. Among PanNET cases, 32–65% have a high degree of T cell infiltrates (CD3 + , CD45RO + , and CD8 +) in intratumoral cases and 50–70%, in extratumoral cases [60]. CD3 +/PD-1^high^ and CD204 +/PD-L1^high^ populations were significantly higher in PanNETs with a higher grade [57]. Furthermore, the expression of PD-L1(Stroma) was higher in PanNEC than in PanNETs [59]. Since immune checkpoints such as PD-1/PD-L1 are mainly expressed in high-grade NEN, the KEYNOTE-028 trial evaluated treatment with pembrolizumab in solid tumors and was able to show that partial response and progression-free survival in patients with PanNETs and PDAC were 6.3% at 4.5 months and 0% at 1.7 months, respectively [61, 62]. Case series have also reported that patients with PanNEC respond to nivolumab and have a long survival of 8 years following standard chemotherapy [63]. Equivalent trials are now in progress for GEP-NETs. However, most phase I and II clinical trials have failed to show any clinical efficacy in the case of PDAC [64].

## Pathological diagnosis and serum detection

The pathology diagnosis of PanNEC is sometimes mistaken for PDAC, as both often have nuclear atypia, necrosis and a high mitotic rate; furthermore, PanNEC may have an adenocarcinoma component [10, 65]. PanNEC is composed of highly atypical neoplastic cells and can be further classified into small and large cell carcinoma. Large cell (61%) is more common than small cell (39%) [66]. In general, PanNEC cells are pleomorphic and its classic salt-and-pepper chromatin pattern is not as evident. Geographic necrosis, as well as perineural and angiolymphatic invasion, are often present in PanNEC. Microscopically, PDAC often has glandular architecture and mucin-producing glands, but can have single-cell infiltration. In addition, the neoplastic cells have irregular nuclear chromatin and prominent nucleoli. Overall, cells in PDAC tend to be more pleomorphic than those in PanNENs. IHC is useful for distinguishing these malignancies. In PanNET, chromogranin A (CgA) and Synaptophysin (Syn) are the most sensitive biomarkers, and staining is generally found to be diffuse and strong; however, CgA and Syn are less sensitive in the diagnosis of PanNEC compared with PanNETs [67]. Well-differentiated morphology and lower Ki67 proliferation index were found to be correlated with the strong expression of SSTR2A and CgA, and highly positive SSTR2A was associated with longer survival in PanNETs [68].

There is overlap in serum biomarkers between PanNEC and PDAC. CA199 and CEA are sensitive and specific markers for PDAC diagnosis and prognosis. Abnormal CA199 and CEA levels have been found in PanNEC, while serum CA199, CEA, and AFP levels above the upper limit have been observed in 23.8%, 28.6% and 19.0% of PanNEC cases, respectively [69]. Abnormal CA199 and CA125 levels are more commonly found in PDAC than in PanNEC. AFP levels in PanNEC are higher than those found in PDAC. Serum CgA has been shown to have the highest sensitivity for diagnosing patients with PanNET G1 and G2. Elevated serum CgA levels have been found in 50–100% of patients with PanNET and have been shown to be correlated with tumor burden and metastasis in PanNETs [70]. However, CgA is less useful for detecting PanNEC tumors, especially in small cell carcinoma [71]. Instead, neuron-specific enolase has been proven to have higher sensitivity; however, its use is limited by its low specificity [72].

## Convergence of clinical management

Standard system therapies vary widely among PDAC, PanNEC and PanNET (Table 2). National Comprehensive Cancer Network treatment guidelines for PanNEC recommend cisplatin and etoposide chemotherapy, with an objective response rate of 41–67% and a median survival of 15–19 months [73]. The standard therapy for PDAC is FOLFIRINOX (5-fluorouracil, irinotecan, and oxaliplatin). FOLIFIRINOX treatment has acceptable side effect profiles and rapid objective response in patients with PanNEC [11]. Somatostatin analogue is standard therapy for PanNETs with dual effects—inhibiting hormone secretion as well as anti-tumor proliferation [56]. The mTOR inhibitor Everolimus is a standard target therapy for PanNETs [74]. The mTOR signaling pathway has also been demonstrated to be active in a small number of PDAC cases, and preclinical data have supported mTORC1 inhibitors in a subgroup of PDAC patients with the hyper-activation of the PI3K-mTOR pathway [75]. However, clinical trials have had disappointing results in PDAC patients treated with Everolimus as a single agent (ORR 0%–6%), or in combination with other agents [76, 77]. The deregulation of the mTOR pathway is not of crucial importance in treatment of PDAC.

**Table 2:** Clinical practices compared between PDAC, PanNEC and PanNET

	PDAC	PanNEC	PanNET
Diagnosis			
Serum test	CA199, CEA,CA125	NSE CA199, CEA, AFP	CgA, 5-HIAA Functional hormone
Imaging	Computer tomography Magnetic resonance imaging F18-FDG PET/CT	Computer tomography Magnetic resonance imaging F18-FDG PET/CT	Computer tomography Magnetic resonance imaging Ga68-DOTATATE PET/CT Somatostatin receptor scintigraphy
Therapy			
Localized	Resection Neoadjuvent chemo + resection	Resection	Active surveillance Resection
Advanced and metastasis	SYSTEM TREATMENT Irinotecan + Oxaliplatin + fluorouracil Gemcitabine + Albumin-Paclitaxel Clinical trials OTHERS Radiotherapy	SYSTEM TREATMENT Etoposide + cisplatin Irinotecan + cisplatin Clinical trials OTHERS Radiotherapy	LOCAL TREATMENT Debulking surgery Transarterial chemoembolization, Ablation of liver metastasis SYSTEM TREATMENT Somatostatin analogs Sunitinib and everolimus Temozolomidecapecitabine
Follow-up	3 months	3 months	Localized 6-12 months; Metastasis 3-6 months

## Conclusions

Current data support the idea that the tumorigenesis of PanNEC and PDAC malignancies might overlap. The genetic profile of PanNEC is distinct from that of PanNETs, while PanNEC harbors TP53 and RB1 alterations, which overlap in PDAC. The genetic profile of PanNEC also lacks neuroendocrine-related genetic changes, such as mutations in MEN1 and ATRX/DAXX. Additionally, a growing body of evidence suggests that there are overlaps in treatments for PDAC and PanNEC, as well as in their prognoses. Our review was able to show an obvious relationship between conventional PDAC and PanNEC in a subset of cases. These preliminary observations raise practical issues for the proper care of patients, which could become the basis for improved therapy. More studies are needed to further discover the still unknown factors surrounding the intersection of PDAC and PanNEC biology and pathology.

## Data Availability

Not applicable.
